# A Single Synonymous Variant (c.354G>A [p.P118P]) in *ADAMTS13* Confers Enhanced Specific Activity

**DOI:** 10.3390/ijms20225734

**Published:** 2019-11-15

**Authors:** Ryan Hunt, Gaya Hettiarachchi, Upendra Katneni, Nancy Hernandez, David Holcomb, Jacob Kames, Redab Alnifaidy, Brian Lin, Nobuko Hamasaki-Katagiri, Aaron Wesley, Tal Kafri, Christina Morris, Laura Bouché, Maria Panico, Tal Schiller, Juan Ibla, Haim Bar, Amra Ismail, Howard Morris, Anton Komar, Chava Kimchi-Sarfaty

**Affiliations:** 1 Hemostasis Branch, Division of Plasma Protein Therapeutics, Office of Tissues and Advanced Therapies, Center for Biologics Evaluation & Research, US FDA, Silver Spring, MD 20993, USA; 2 Gene Therapy Center, University of North Carolina at Chapel Hill, Chapel Hill, NC 27599, USA; 3 Present Address: Department of Emergency Medicine, Banner University Medical Center, The University of Arizona, Tucson, AZ 85724, USA; 4 BioPharmaSpec Ltd., St. Saviour JE2 7LA, UK or or; 5 Department of Life Sciences, Imperial College London, South Kensington Campus, London SW7 2AZ, UK; 6 Present Address: Antikor Biopharma Ltd., Stevenage Bioscience Catalyst, Gunnels Wood Road, Stevenage SG1 2FX, UK; 7 Departments of Cardiac Surgery and Anesthesiology, Perioperative and Pain Medicine, Boston Children’s Hospital and Harvard Medical School, Boston, MA 02115, USA; 8 Department of Statistics, University of Connecticut, Storrs, CT 06269, USA; 9 Center for Gene Regulation in Health and Disease, Department of Biological, Geological & Environmental Sciences, Cleveland State University, Cleveland, OH 44115, USA

**Keywords:** ADAMTS13, synonymous variant, translation, codon usage, specific activity, post-translational modifications, ribosome profiling

## Abstract

Synonymous variants within coding regions may influence protein expression and function. We have previously reported increased protein expression levels ex vivo (~120% in comparison to wild-type) from a synonymous polymorphism variant, c.354G>A [p.P118P], of the *ADAMTS13* gene, encoding a plasma protease responsible for von Willebrand Factor (VWF) degradation. In the current study, we investigated the potential mechanism(s) behind the increased protein expression levels from this variant and its effect on ADAMTS13 physico-chemical properties. Cell-free assays showed enhanced translation of the c.354G>A variant and the analysis of codon usage characteristics suggested that introduction of the frequently used codon/codon pair(s) may have been potentially responsible for this effect. Limited proteolysis, however, showed no substantial influence of altered translation on protein conformation. Analysis of post-translational modifications also showed no notable differences but identified three previously unreported glycosylation markers. Despite these similarities, p.P118P variant unexpectedly showed higher specific activity. Structural analysis using modeled interactions indicated that subtle conformational changes arising from altered translation kinetics could affect interactions between an exosite of ADAMTS13 and VWF resulting in altered specific activity. This report highlights how a single synonymous nucleotide variation can impact cellular expression and specific activity in the absence of measurable impact on protein structure.

## 1. Introduction

Contrary to the historical belief that synonymous variants are biologically inert, the potential for such variants to impact protein expression and function and underlie human disease is increasingly recognized [1,2,3,4,5,6]. To date, nearly 50 distinct diseases affecting various organ systems have been described in association with synonymous variants [4]. Synonymous variants falling within consensus splice sites may drastically alter protein composition and functionality [7]. Coding synonymous variants can also impact protein biogenesis in many other appreciable ways. Protein translation accuracy and efficiency are mechanistically linked to codon usage [8,9,10]. Synonymous codon substitutions can influence the translation kinetics and the timing of co-translational protein folding [3,4,7,11].

ADAMTS13 (a disintegrin and metalloproteinase with a thrombospondin type 1 motif, member 13) is a large multi-domain secreted protein that regulates thrombogenesis by cleavage of an adhesive blood glycoprotein, von Willebrand factor (VWF) [12,13]. Classically, this enzyme has been studied for its association with thrombotic thrombocytopenic purpura (TTP), a life-threatening hematological disease directly linked to severe ADAMTS13 deficiency. A growing list of additional types of pathologic thrombosis may be influenced by this protease, including stroke and myocardial infarction [12]. A prospective study of nearly 6000 participants in the Rotterdam cohort found individuals falling in the lowest quartile of ADAMTS13 activity have a 7.3% absolute increased risk of ischemic stroke over those in the highest quartile [14]. Much investigative attention has been directed towards the significance of varying degrees of ADAMTS13 deficiency. However, ADAMTS13 activity varies widely, both positively and negatively, across the general population [15]. The implications of elevated ADAMTS13 function are uncertain. Increased levels of ADAMTS13 can represent a compensatory response to persistently elevated levels of VWF in patients with venous thromboembolism [16]. Alternatively, altered synthesis or enzymatic activity can result from variants in the *ADAMTS13* gene [17,18].

In previous research [18], we have investigated the effects of six synonymous and six non-synonymous variants found in *ADAMTS13* on ADAMTS13 expression and function We have used in silico approaches and ex vivo transient expression system in mammalian cells to study the effects of these substitutions on mRNA structure/stability, protein expression levels, and enzymatic activity [18]. In the course of these studies, we found that a single synonymous variant at a proline residue within the metalloprotease domain, c.354G>A [p.P118P] (rs28571612), displayed higher cellular expression levels in transient transfection experiments, compared to that of the wild-type (WT) *ADAMTS13* [18]. This substitution is a naturally occurring variant in the human population (allele frequency: 0.026 (1000 Genomes) [19], 0.0627 (ExAC) [20]), and has not been associated with a disease phenotype. Naturally occurring genetic variants that augment protein expression hold promise for bioengineering purposes, but a thorough evaluation of their effects on physical and functional properties of the encoded protein is required. Moreover, in the last few years more than 50 diseases were reported to be associated with a single synonymous mutation in the disease associated gene [4]. Usually, these mutations decrease protein expression levels and/or activity, therefore, leading to a disease [4]. However, c.354G>A variant represent a unique example in this regard in which the synonymous variation produces a substantial positive contribution to protein expression levels [18]. Therefore, we decided to extensively characterize c.354G>A variant and compare its properties to that of the WT *ADAMTS13*. We set out to interrogate the effects of c.354G>A variant on translation and physicochemical properties of the ADAMTS13 protein by assessing in silico parameters, translation kinetics, specific activity, structural conformation, and post-translational modifications by employing in silico tools, in vitro cell free translation system, ribosome profiling, and purified proteins from lentiviral stable transfection system or Flp-In system, which assure a single copy at a single integration site.

## 2. Results

### 2.1. In Vitro Translation Assay Revealed Higher Protein Yield From c.354G>A [p.P118P] Variant

**Figure 1:**
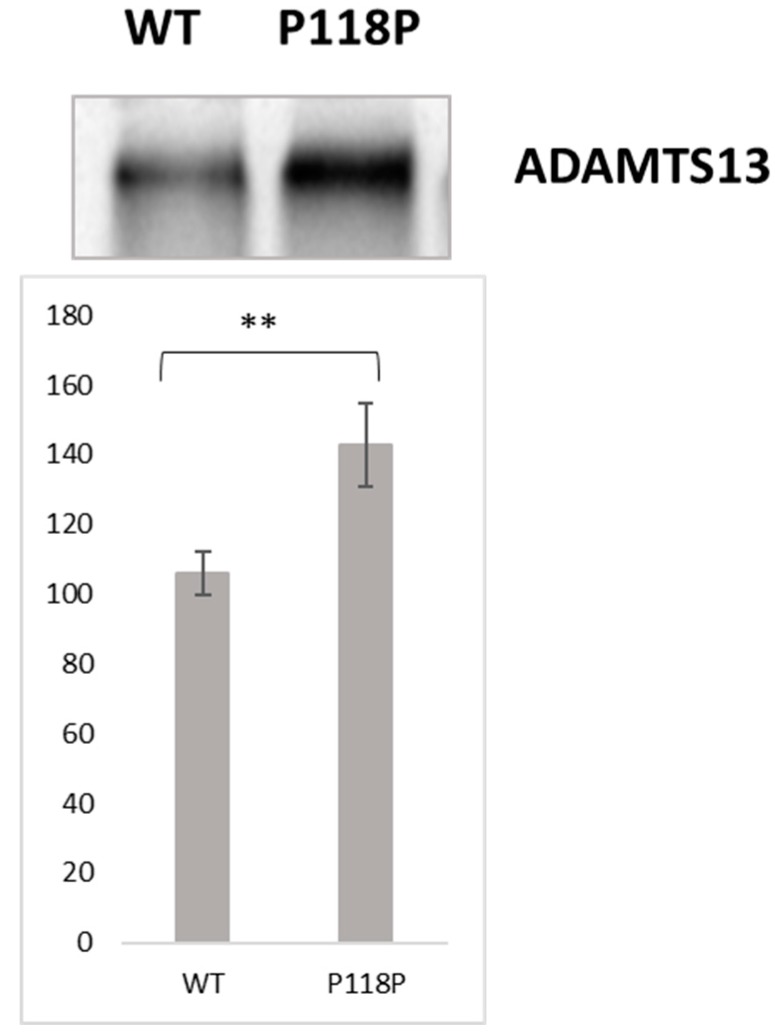
In Vitro translation analysis of wild-type (WT) and c.354G>A variant *ADAMTS13* transcripts. Top—representative autoradiogram of WT and p.P118P (c.354G>A) variant in vitro translation products. Bottom—the graphical representation of intensities of in vitro translation products. ** *p* < 0.005 by Student’s *t*-test (Graphpad), Data represents the mean ± SEM of three independent experiments. Significantly higher translation of c.354G>A variant relative to WT transcript was observed.

Due to the differences in the extracellular expression of p.P118P (c.354G>A) and WT ADAMTS13 in transient transfection experiments found in our earlier study [18], we applied in vitro translation assay and an evolving technique named ribosome profiling to determine if this single-codon difference would alter the translation kinetics of *ADAMTS13* transcript. In vitro translation assay compares translation efficiency in an isolated system using cell-free extracts to carry out translation. In vitro translation of the p.P118P transcript, revealed enhanced (~1.4 fold) translation of the p.P118P (c.354G>A) variant relative to WT (Figure 1 and Figure S1).

This result suggests that enhanced expression of ADAMTS13 from p.P118P variant observed ex vivo is likely a result of the enhanced translation of the p.P118P (c.354G>A) variant. Ribosome profiling was first described in 2009 by Ingolia and Weissman [21] and involves the use of next generation sequencing to identify ribosome protected fragments of mRNA, providing a nucleotide-resolution map of translating ribosome throughout the transcriptome. We used ribosome profiling data to assess codon level distribution of ribosome footprints and translation efficiency, measured as a ratio of ribosome-protected fragments to mRNA fragments for both the WT and p.P118P transcripts. Our data showed average translation efficiency values of 1.299 for WT and 1.277 for p.P118P, revealing no significant differences. Examination of the cumulative sum of the normalized log (Ribosome Protected Fragments) of WT and p.P118P transcripts and normalized ribosome profiling read counts per codon also did not reveal significant differences (Figures S2 and S3). The lack of differences observed in ribosome profiling experiment seems to be because of ribosome profiling as applied here only captures a snapshot of ribosome distribution and does not directly determine translation speed.

### 2.2. In Silico Analysis of c.354G>A Variant Revealed That This Mutation Leads to the Introduction of a More Frequently Used Codon/Codon Pair(s)

Synonymous variants alter the local codon usage, which can potentially lead to changes in translation kinetics. Frequently used codons were suggested to be translated faster than rare codons and used at a greater frequency in highly expressed genes, albeit this association was found to be weaker in mammals compared to lower order organisms [22,23]. Additionally, codon pair bias referring to non-random occurrence of adjacent codons within open reading frames was also shown to affect translation, thereby affecting expression levels [24]. Due to the enhanced expression of ADAMTS13 from c.354G>A variant in transient transfection experiments and in vitro translation assay, we assessed the alterations in local codon usage characteristics in c.354G>A variant in comparison with WT sequence by measuring relative synonymous codon usage (RSCU) and codon pair frequencies.

RSCU is traditionally defined as the frequency of observed codons divided by expected if usage of all synonymous codons for a particular amino acid was uniform [25]. RSCU values range from 0 and the number of codons for a particular amino acid. A value of 1 indicates the lack of bias. Proline is encoded by four synonymous codons; CCG, CCA, CCC, and CCT. Here, we calculated the RSCU values of prolineencoding codons in the entire human genome and solely within the *ADAMTS13* cDNA (Table 1). This analysis revealed that the CCG codon coding for proline 118 in the WT sequence is underrepresented in *ADAMTS13* as well as across the entire human genome, and that any synonymous substitution from CCG (WT) to CCA (variant under study), CCC, or CCT will shift the frequency of the usage of the codon at this position to higher values (more frequently used proline codon in *ADAMTS13* and the entire human genome). Since the liver is the major source of ADAMTS13 [26], we have also calculated RSCU values based on liver tissue specific codon usage frequencies. RSCU values from this analysis were similar to and in agreement with those obtained from *ADAMTS13* and genome wide codon usage frequencies (Table 1). Therefore, substantial increase in RSCU was seen for synonymous variant c.354G>A [p.P118P] (Table 1).

Furthermore, we have compared codon pair (codon context) usage patterns of proline codons in the context of preceding and following codons (GAC and TCC, respectively) in WT *ADAMTS13* sequence and the c.354G>A [p.P118P] variant. Due to the very high number of possible codon pairs (4096), it is reasonable to perform this analysis for human genome and liver tissue specific codon pair usage, but not for *ADAMTS13* alone. Analysis of human genome codon pair usage revealed increased percentile shifts (48th to 84th percentile, and 35th to 72nd percentile, respectively, for the preceding and following codon pair) for the c.354G>A variant over the WT sequence (Table 2). The other two synonymous codons for proline, CCC, and CCT also showed similarly high codon pair usage percentiles (CCT with 88th and 81st percentile scores for preceding and following codon pairs, respectively; CCC with 94th and 86th percentile scores for preceding and following codon pairs, respectively). Similar patterns were seen in liver tissue, where the WT sequence had codon pair frequencies under the 30th percentile, while all c.354G>A/C/T variants yielded significantly higher percentile ranks (Table 2). Overall, these results show that c.354G>A substitution resulted in the introduction of frequently used codon/codon pair(s) relative to WT sequence, and this in turn could have potentially led to enhanced translation of the c.354G>A [p.P118P] variant.

Since altered translation kinetics can affect co-translational folding and protein conformation [27,28], we have proceeded to assess the impact of enhanced translation and expression from c.354G>A [p.P118P] variant on ADAMTS13 protein folding/conformation, post-translational modifications and specific activity.

### 2.3. Limited Proteolysis and Circular Dichroism Analysis of the Wild-Type and p.P118P Variants Did Not Identify Conformational Differences

**Figure 2:**
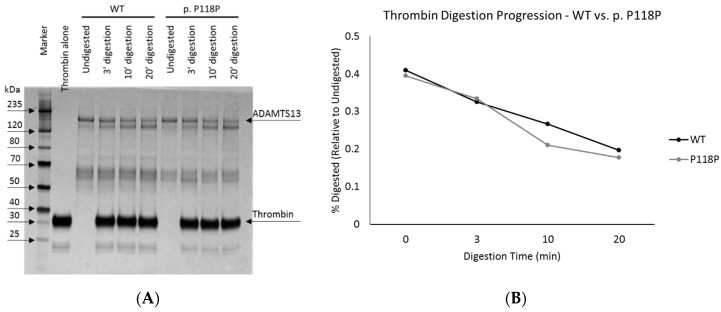
Thrombin digestion of wild-type (WT) and p.P118P variant ADAMTS13. Panel (**A**) shows the silver stained gel image of WT and p.P118P variant ADAMTS13 digested by thrombin for 3, 10, and 20 min. Panel (**B**) shows graphical representation of intensities of top two bands. No significant differences were observed.

We performed enzymatic digestion and circular dichroism (CD) analysis of the purified protein to assess conformational differences between WT and p.P118P variants. Subtle differences in protein conformation can be inferred from differential access of a digestive enzyme to cleavage sites within a substrate protein. We have employed thrombin, an enzyme with known proteolytic activity against ADAMTS13 [29], for this analysis. In this assay, digestion of purified WT and p.P118P variants with thrombin for 3, 10, or 20 min, revealed no significant differences (Figure 2). Similarly, CD analysis also revealed largely similar spectral profiles revealing no significant differences (Figure S4).

### 2.4. Post-Translational Modifications Analysis of Wild-Type and p.P118P Variants Showed No Significant Differences, but Revealed Previously Unreported Modifications

**Figure 3:**
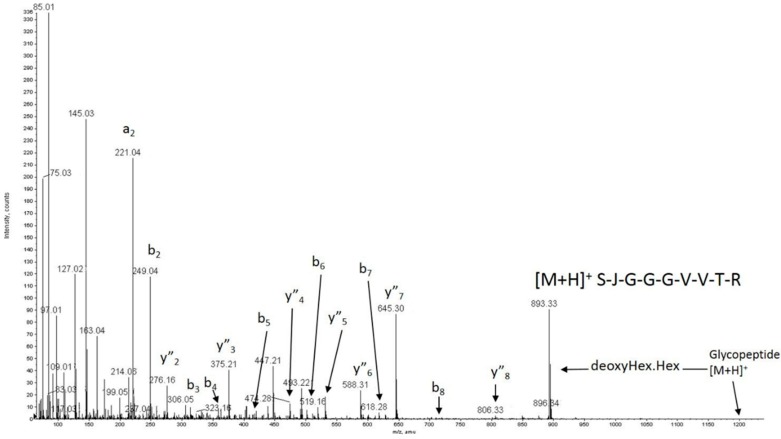
The MS/MS spectrum of m/z 601.22+ showing (i) the b and y″ ions used to assign the sequence within ADAMTS13 WT (as SJGGGVVTR), and (ii) the facile loss of 308.1 Da from the quasimolecular ion to give m/z 893.3 corresponding to the loss of a DeoxyHex.Hex disaccharide. Data from the MS (low energy) spectrum show an initial loss of Hexose followed by Deoxyhexose, confirming the substitution order expected for GlucosylFucose at the JXXSJG consensus site (here JSRSJG) seen in other TSRs [41]. J = Carboxymethyl Cysteine.

Using mass spectrometry, we also assessed post-translational modifications, which are well recognized to impact protein secretion and protein folding [30]. The data collected were interpreted according to the standard principles of protein and glycoprotein mass spectrometric fragmentation pathways [31,32,33]. The mass spectrometry data for WT ADAMTS13 demonstrated the presence of seven Thrombospondin Repeats (TSRs; 1, 2, 3, 5, 6, 7, and 8) possessing the consensus sequences for possible *O*-fucosylation of S/T [34,35], all seven of which were found to be modified with deoxyhexose-hexose in the present study (Table 3). Interestingly, the data showed clear evidence for the presence of the previously unreported *O*-glycosylation of TSR-1 at S399 (Figure 3).

Additional analysis of WT ADAMTS13 indicated the glycosylation of a single tryptophan residue with a hexose sugar (Figure S5). This residue was identified as W387 as opposed to the previously published putative C-mannosylation site at W390 [36]. The MS/MS spectrum of the tryptic peptide showed a WSSWGPR sequence (which satisfied the recognition motif WXXW for protein C-mannosylation) and confirmed the addition of the hexose sugar to the first tryptophan residue. This consensus sequence has been predicted in nearly all members of the ADAMTS superfamily and C-mannosylation has been described in Thrombospondin-1, punctin-1, and ADAMTS5 [37,38]. An earlier study of an electron density map of a recombinant disintegrin (D), thrombospondin type-1 repeat-1 (T), cysteine-rich (C), and spacer (S) domains (“DTCS”) fragment of ADAMTS13 suggested the possible C-mannosylation of W387 [39] and W387 was recently suggested to be the preferred site of C-mannosylation of TSR1 in plasma derived ADAMTS13 [40]. To our knowledge, this is the first time this post-translational modification has been rigorously identified in recombinant ADAMTS13. Interpreting the principal fragment ions observed in the MS/MS spectrum in Figure S5 suggests the novel formation of a 2-Ethynyl-Indole (Acetylenic substituent) on the W387 side chain. This results, as shown, from the loss from the quasimolecular ion 519.2^2+^ of 138 Da due to water losses and partial cleavage of the hexose ring, to give m/z 450.2^2+^ in preference to the normal β-elimination (162 Da) seen in *O*-linked glycosylation chemistry (Figure S5). Further, a previously unreported *O*-glycosylation of WT ADAMTS13 was found at S1170, which resides on the border of the TSR 1–8 and CUB1 protein domains. As seen in Figure S6, this post-translational modification was defined as DiSialyl Core-1 (NeuAc_2_HexHexNAc) and a lesser amount of NeuAc_2_Hex_2_HexNAc_2_ was also present. The specific roles of these newly identified post-translational modifications remains to be determined.

Importantly, the above site-specific post-translational modifications found in WT ADAMTS13 were also found in p.P118P variant (Table 3). While slight differences were observed in, for example, the amount of additional hexosylation (to give Fuc-Hex_2_) or fucosylation alone at some positions, or in the relative amount of the hexasaccharide compared to the DiSialyl Core-1 pentasaccharide found at S1170 in WT ADAMTS13, the overall degree of post-translational modifications at the residues observed was found to be very similar for both samples.

### 2.5. FRETS-VWF73 Assay Revealed Increased Specific Activity of p.P118P Variant

**Figure 4:**
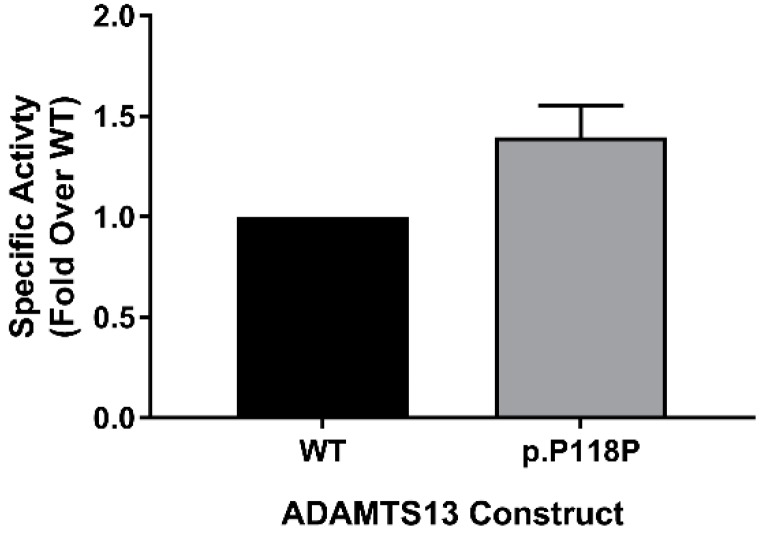
Specific activity assessment. Activity of equimolar concentrations of wild-type (WT) and p.P118P variant ADAMTS13 was measured by FRETS-VWF73 activity assay. Specific activity of p.P118P variant was higher than WT ADAMTS13. Each bar (*n* = 3) represents mean activity (±standard deviation) calculated as fold change over WT.

Using FRETS-VWF73 assay [42], we measured the activity of purified WT and p.P118P variants to assess the potential impact of altered translation on specific activity. We have measured activity in purified protein samples from two independent preparations that were ensured to have equal concentrations by densitometry analysis of western blots (silver staining and antibody probing). In this analysis, rather unexpectedly, p.P118P variant showed higher specific activity over WT (1.4 ± 0.16-fold, *p* = 0.0349) (Figure 4). This observed difference was in spite of lack of discernible changes in conformation or post-translational modifications.

### 2.6. A Combination of Subtle Conformational Changes Arising from c.354G>A Variant and the Proximity of Proline 118 to an Exosite in Disintegrin Domain May Explain Increased Specific Activity

**Figure 5:**
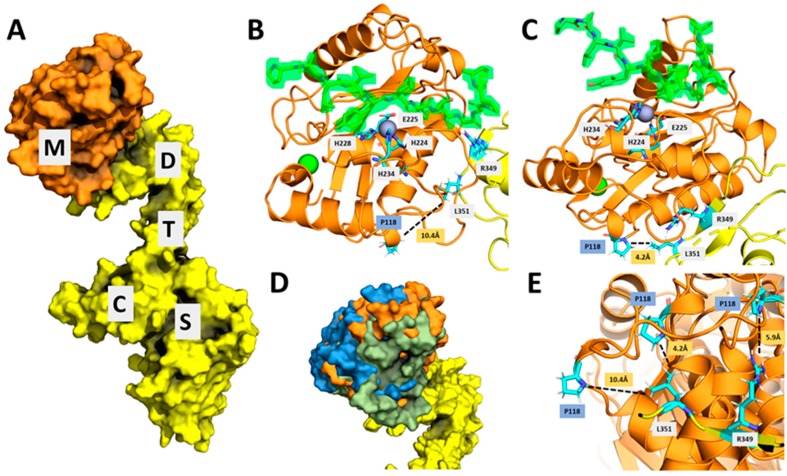
Predicted interactions of homology model of metalloprotease (M) domain and crystal structure of DTCS (Crystal structure of Disintegrin (D), TSR Repeat-1 (T), Cysteine-rich (C), and Spacer (S) domains of ADAMTS13) domains. (**A**) A top-scoring model of the metalloprotease (M, orange) docked (using Rosetta Dock Protocol) onto the DTCS domains (yellow) from ADAMTS13. (**B**) Model showing the active site of the metalloprotease including the Zn ion (grey sphere), Ca ion (green sphere), active site residues (H228, H224, H234, E225), synonymous variant p. P118P, VWF A2 cleavage peptide PALVYMVTGNPA (green highlight), and previously reported important residues in the D domain (L351, R349). Distance between P118 and D domain residues (dotted line) is measured throughout to demonstrate the movement of possible different conformational states as the metalloprotease domain interaction with the D domain changes upon previously demonstrated allosteric. (**C**) Another top-scoring model shows that the metalloprotease is in a different conformation relative to the D domain, changing the distance between the P118 and the L351. (**D**) Three top-scoring models (blue, orange, and green) aligned on top of each other show the different conformational space the metalloprotease can be docked relative to D domain. (**E**) The three top-scoring models (same as D) are aligned and shown with the different distances between P118 and the L351 and R349 residues on the D domain.

The mature ADAMTS13 is composed of metalloprotease (M), disintegrin (D), thrombospondin type-1 repeat-1 (TSR-1), cysteine-rich (C), spacer (S), TSR2–8, and CUB1-2 domains in the same order from N-terminus [12]. When the TSR2–8 and CUB domains are excluded, the resulting protein is generally described as MDTCS and when the metalloprotease is missing, it is labeled as DTCS. The metalloprotease domain contains a Zn^2+^ active site responsible for substrate cleavage in which the Zn ion helps stabilize the charge generated on the carbonyl carbon during VWF A2 cleavage. The disintegrin, cysteine-rich, and spacer domains contain an exosite each that are important for substrate binding and specificity [43]. From multiple sources, it is clear that the ADAMTS13 molecule undergoes many conformational changes in order to bind and cleave its substrate, VWF [44,45]. Yu et al. [44] employed atomic force microscopy and demonstrated that the ADAMTS13 exists in multiple conformational states and the intra-domain interactions could be altered, thus affecting the flexibility of the ADAMTS13 molecule. Zhu et al. [45] used small-angle x-ray scattering (SAXS) to determine that the ADAMTS13 folds in half to allow formation of an allosterically regulated MDTCS complex. The folding allows for the multidomain protein to be autoinhibited by the TSR and CUB domains. In addition, the electron density envelopes (SAXS data) of the ADAMTS13 protein with the TSR domains removed showed conformational changes of the metalloprotease relative to the D domain. These allosteric forces are self-regulated either by self-inhibition or binding to the VWF. We hypothesized that altered translation kinetics arising from altered codon usage of c.354G>A [p.P118P] variant resulted in subtle conformational changes that are not readily identifiable by limited proteolysis assay but led to increased specific activity. To investigate this, we have examined the proximity of proline 118 located in the metalloprotease domain to the functionally important sites (active site and exosites) within ADMATS13. Since crystal structure of metalloprotease domain in not available [43], we docked a predicted structure of the metalloprotease domain with the crystal structure of the DTCS (PDB 3GHM) domains [46], resulting in many different conformations. In Crawley et al., 2011 [47], the predicted interface between the metalloprotease and VWF also includes part of the disintegrin domain. Some of the resulting conformations show the metalloprotease and disintegrin domains similarly aligned. Other conformations, however, show the proline at position 118 located on the surface and to be near the disintegrin domain, with the metalloprotease and disintegrin domains improperly aligned (Figure 5). Within disintegrin domain, arginine and leucine at positions 349 and 351, respectively function as exosite and are important for the ADAMTS13 proteolytic activity [48]. Some of the top predicted models in our study indicated the proximity (<10 Å) of proline 118 to the leucine 351 and arginine 349 in disintegrin domain. These results suggested the possibility of subtle conformational changes resulting from altered translation kinetics of p.P118P variant affecting the exosite and VWF interactions and improved specific activity.

## 3. Discussion

Synonymous variants have long been assumed to lack relevance and are often ignored owing to the belief that they have little to no effects on protein expression and function. Consequently, the structural and functional characteristics of non-synonymous mutations are widely investigated while studies of synonymous mutations have been few in comparison. Perhaps the best understood mechanism by which synonymous mutations impact cell biology is through alterations of consensus splice sites [7]. However, in recent years it has become apparent that synonymous variants can affect the expression, structure, and functionality of proteins through multiple other mechanisms, which in particular could include their impact on kinetics of protein translation [1,7,49,50,51]. This notion is reflected by the growing body of work linking synonymous mutations to human disease [4].

In this research we set out to characterize a naturally-occurring synonymous variant at the proline 118 position in the *ADAMTS13* gene, a genetic variant that we previously found to produce increased protein expression [18]. We first assessed the effect of c.354G>A [p.P118P] variant on the translation kinetics using both in vitro translation and ribosome profiling. Cell free in vitro translation revealed enhanced (~1.4-fold) translation for c.354G>A [p.P118P] variant in comparison with WT. Such difference, however, was not recapitulated by ribosome profiling, where no difference in calculated translation efficiency between the WT and p.P118P transcript was found. This discrepancy likely stems from technical differences between cell-free in vitro translation and ribosome profiling, as ribosome profiling as applied in this research does not directly measure translation speed. We considered potential mechanisms to explain increased protein expression of the p.P118P variant by analyzing codon and codon pair frequency data. This revealed that c.354G>A substitution resulted in the introduction of a more frequently used codon and the increased percentile shifts (48 → 84, 35 → 72) of codon pairs between the WT and the synonymous variant for both the preceding and following codon pair. The higher frequency codon and codon pair data for this proline variant may have improved translation efficiency of c.354G>A [p.P118P] variant [52,53].

Protein folding is a dynamic process involving complex interactions between ribosomes and molecular chaperones and is affected by translation elongation kinetics [54]. Enhanced translation rate of p. P118P (c.354G>A) variant could affect ribosome–chaperone interactions, thereby co-translational folding and lead to changes in protein conformation [27,28]. Subsequently, we assessed the effect of altered translation kinetics on p.P118P variant structure and additionally on post-translational modifications and specific activity. In this analysis, we unexpectedly found that the p.P118P variant possesses higher specific enzymatic activity. This finding could not be explained by any differences in post-translational modifications found on WT ADAMTS13 as compared to the p.P118P variant. Moreover, thrombin digestion kinetics of the purified WT and p.P118P ADAMTS13 protein were nearly identical, likely indicating overall very similar protein conformations. Based on these results, we hypothesized that altered translation kinetics of p.P118P variant may have resulted in subtle conformational differences that are not readily identifiable, but altered specific activity. In structural analysis, modeled interactions between predicted metalloprotease domain structure and crystal structure of DTCS domains revealed the proximity of P118 located in metalloprotease domain to L351 and R349 that function as exosite in disintegrin domain. It has been shown that residues R349, L350, L351, and V352 enhance the binding of the metalloprotease to the Tyr1605–Met1606 scissile bond in the A2 domain [48]. These results indicated the possibility of subtle conformational differences in p.P118P variant affecting the interactions between exosite in disintegrin domain and VWF substrate, thereby specific activity. The ADAMTS13 protein samples of WT and p.P118P variants used in conformational and specific activity assessment were purified from Flp-In 293 cells, which stably express protein of interest from single copy per cell. Therefore, in the context of cellular proteome that is being actively translated in these cells, rather than the overall increase in the amount of protein synthesis, changes in the local translation kinetics of p.P118P variant could play an important role in predicted conformational changes.

Synonymous coding variants that are capable of bolstering protein expression are of increasing interest in the development of biotherapeutics as they can increase the cellular yield of therapeutic recombinant proteins. Over the past decade advancements in synthetic gene synthesis have allowed for the application multiparametric algorithms to optimize native coding sequences. These technologies take advantage of the degeneracy of the genetic code and typically employ artificial synonymous codon substitutions, which can have unpredictable effects on local protein translation kinetics and protein function. The use of non-pathogenic, naturally occurring synonymous variants that are recognized to augment protein expression may be an alternative strategy to achieve improved expression characteristics. While a single codon substitution would not generally be expected to lead to a meaningful change in protein translation efficiency, the combination of multiple such variants in a single synthetic gene could have pleotropic effects on recombinant protein production and function.

## 4. Materials and Method

### 4.1. Cell Line Establishment and Maintenance

Human embryonic kidney cells (HEK293T; ATCC, Manassas, VA, USA) and Flp-In 293 cells (Thermo Fisher Scientific, Waltham, MA, USA) were employed for the generation of stable expression cell lines. Both cell lines were grown in Dulbecco’s Modified Eagle Medium (Quality Biological, Inc., Gaithersburg, MD, USA) with 10% fetal bovine serum (Thermo Fisher Scientific, Waltham, MA, USA) at 37 °C under humid conditions in 5% CO_2_.

HEK293T cells stably expressing WT (NM_139025.4) and p.P118P variants were established following transduction with lentiviral vectors, as previously described [55]. These cell lines expressed ADAMTS13 and green fluorescent reporter protein from single mRNA transcript using an IRES (internal ribosome entry site) element from encephalomyocarditis virus (ECMV). To establish heterogeneous cell populations, HEKT293T cells were transduced with the lentiviral vectors at m.o.i < 0.1 and selected in the presence of 60 µg/mL blasticidin (InvivoGen, San Diego, CA, USA). GFP expression was quantified via flow cytometry and Western blot to ensure equivalent transduction efficiency.

Flp-In 293 cells enable the homogeneous expression of a gene of interest from a single copy per cell at a specific genomic location. Flp-In 293 cells stably expressing the WT and p.P118P variants were generated following manufacturer’s instructions. Briefly, Flp-In 293 cells were transfected with pcDNA5/FRT vectors encoding full-length cDNA of WT or p.P118P variant and the pOG44 vector. Stably transfected cells were selected in the presence of 200 µg/mL hygromycin.

Both HEK293T and Flp-In 293 stable expression cell lines expressed c-terminal V5-His tag fusion versions of WT and p.P118P variants under the control of a CMV promoter. The ADAMTS13 protein purified from Flp-In 293 cells was used for the assessment of specific activity and structural conformation analysis of the WT and p.P118P variants. The ADAMTS13 protein purified from relatively high expression lentiviral transduced HEK293T cells was employed for the post-translational modification analysis of WT and p.P118P variants.

### 4.2. ADAMTS13 Purification and Quality Assessment

Purification of extracellular secreted WT and p.P118P variant ADAMTS13 was accomplished by centrifugation of raw conditioned medium in 10 kDa molecular weight cut off (MWCO) protein concentrators (MilliporeSigma, Burlington, MA, USA) followed by affinity tag purification against the C-terminal V5 epitope using an anti-V5-tag resin (MBL International Corporation, Woburn, MA, USA). ADAMTS13 was eluted from the resin by excess V5 peptide in 1X PBS. The V5 peptide was subsequently diluted from the sample by repeated application of the sample in 10 kDa MWCO centrifugal devices (MilliporeSigma, Burlington, MA, USA). Purity and equal concentration of WT and p.P118P variant ADAMTS13 was ensured by densitometry analysis following silver staining (Invitrogen) and Western blot probing with rabbit anti-V5 monoclonal antibody (ab206566, Abcam, Cambridge, MA, USA). Goat anti-rabbit HRP (ab6721, Abcam, Cambridge, MA, USA) was used as a secondary antibody. A chemiluminescent signal was developed using Super Signal West Pico Chemiluminescent Substrate (Thermo Fisher Scientific, Waltham, MA, USA) and images were generated using a Kodak Image Station 4000 MM Pro (Carestream Health, Rochester, NY, USA). Densitometry analysis was performed with Carestream Molecular Imaging Software (version 5. 0. 6. 20, Carestream Health, Rochester, NY, USA).

### 4.3. In Vitro Translation Assay

In vitro translation assay was performed as described earlier [7]. Briefly, the assay was performed with rabbit reticulocyte lysate (RRL) cell-free system (Cat # L4960, Promega, Madison, WI, USA) supplemented with calf liver tRNAs as per manufacturer’s instructions. In vitro transcription was performed using mMessage mMachine T7 Ultra Transcription kit (Cat # AM 1345, Thermo fisher Scientific, Waltham, MA, USA). Capped mRNAs were purified by lithium chloride precipitation method (as per manufacturer’s instructions; Cat # AM 1345). For transcription, the plasmids were linearized with BclI. This gives rise to mRNA products, harboring 14 nt past the stop codon. Dittmar et al. [56] reported an association between tissue-specific tRNA abundances and codon usage. In our analysis, the codon usage frequencies of human [57] and bovine liver tissue [58], source of tRNAs, were largely similar and indicated the suitability of our system to study the translation kinetics of WT and c.354G>A variants.

### 4.4. Ribosome Profiling—Library Preparation and Data Processing

Library preparation and data processing for ribosome profiling experiment was performed as described previously [59]. HEK293T cells with stably integrated WT and c.354G>A variants of *ADAMST13* were harvested at ~80% confluence following overnight incubation in Opti-MEM to maintain consistency with protein level processing and analyses presented in this paper. Ribosome profiling was conducted using the Illumina TruSeq Ribo Profile (Mammalian) Kit (Illumina, San Diego, CA, USA) according to the manufacturer’s instructions with modifications in harvest, RNA isolation/purification (isopropanol isolation used to improve yield), and ribosome-protected fragment (RPF) selection (~20–32 nt). During harvest, media was carefully removed, and cells were immediately flash-frozen. Cells were quickly scrapped into 1 mL of ice-cold lysis buffer (5X Mammalian Polysome Buffer, 10% Triton-X100, 100 mM DTT, DNase I, Nuclease-free water) and homogenized on ice by passing through a 26G needle 10 times. Lysate was then spun at 4 °C for 10 min at 20,000× *g*. Supernatant was aliquoted into cryovials and immediately frozen in liquid nitrogen for future use. Samples were sequenced using Illumina HiSeq 2500 (Illumina, San Diego, CA, USA). For each construct, libraries were prepared from two biological replicates of three technical replicates each, for a total of six experimental runs.

Sequencing data was pre-processed by adapter trimming (FASTX Toolkit, version 0.0.14, http://hannonlab.cshl.edu/fastx_toolkit/, Cold Spring Harbor, NY, USA) and removal of contaminating rRNA and tRNA sequences using bowtie version 1.0.0 (seed length—1 set to 20 and all other parameters default, http://bowtie-bio.sourceforge.net/index.shtml, JHU, Baltimore, MD, USA). Using TopHat version 2.0.9 (options—no-novel-juncs to only consider junctions in a given GFF file and g 20 for a maximum number of 20 multiple alignments per read, all other parameters default), (https://ccb.jhu.edu/software/tophat/index.shtml, JHU, Baltimore, MD, USA), RIBO-SEQ and RNA-SEQ populations were aligned to a custom human transcriptome built comprised of 19,634 transcripts using the GENCODE hg19 protein coding sequence and untranslated region annotations. Fragments smaller than 25 nucleotides in length were removed from the RNA-SEQ samples and fragments of 20–22 and 28–30 nucleotides in length were isolated from RIBO-SEQ samples. A P-site offset of 12 nucleotides from the 5′ end of the fragment was used for RIBO-SEQ footprints. This was determined by analyzing the distribution of footprints by their size and distance from the start codon of the 5′ end. All code for data processing was written in Python 2.7 (https://www.python.org/, Python Software Foundation, Wilmington, DE, USA).

Ribosome profiling data for *ADAMTS13*, *GAPDH*, and *ACTB* are plotted as the average number of normalized reads with codon c in the (A) site of the ribosome. Normalized reads were calculated as

(1) $$\#\;Normalized\;Reads\;c=\frac{N\;Reads\;c}{\left(N\;Reads\;CDS\;÷Length\;CDS\right)}$$

Translation efficiency (TE) for WT and p.P118P transcripts was calculated as TE = RPF (RPKM)/mRNA (RPKM).

### 4.5. Calculation of Codon and Codon Pair Usage Characteristics

RSCU was calculated for prolineencoding synonymous codons using the method of Sharp and Li [60]. Each codon was analyzed relative to both the codon frequency within *ADAMTS13* cDNA as well as the entire human genome. The codon and codon pair usage frequencies of human genome were obtained from HIVE-CUTS and Codon and Codon-Pair Usage Tables (CoCoPUTs, https://hive.biochemistry.gwu.edu/cuts/about) [57,61,62]. Tissue specific codon and codon pair usage of liver were calculated from human codon and codon pair usage by CDS derived from CoCoPUTs and liver-specific transcriptomic data (transcripts per million) derived from the Genome Tissue Expression (GTEx) Portal V7 (dbGaP Accession phs000424.v7.p2, https://gtexportal.org/home/index.html) [63] (both databases accessed on 15 July 2019). Scripts to calculate liver-specific codon pair usage were written in Python 2.7.

### 4.6. Limited Proteolysis—Thrombin Digestion

To assess the conformational differences between WT and p.P118P variants, limited proteolysis with thrombin (MilliporeSigma, Burlington, MA, USA) was performed as described previously [29]. Briefly, ADAMTS13 and thrombin proteins were resuspended in reaction buffer comprising of 150 mM NaCl, 5 mM CaCl_2_, and 20 mM Tris. Equimolar concentrations of WT and p.P118P ADAMTS13 were incubated with thrombin at 37 °C for 3, 10, or 20 min. Reaction was stopped by the addition of 4X LDS buffer with DTT (Thermo Fisher Scientific, Waltham, MA, USA) and boiling of samples for 20 min at 100 °C. Digestion pattern of ADAMTS13 proteins was assessed by silver staining of SDS-PAGE gels (Thermo Fisher Scientific, Waltham, MA, USA).

### 4.7. Circular Dichroism (CD) Analysis

CD analysis was performed as described previously [49]. Briefly, CD spectra were measured using Jasco J-810 CD Spectrophotometer (Jasco, Oklahoma City, OK, USA). Equal concentration protein samples were placed in 1 mm path length cells and measurements were taken at a constant temperature of 5 °C and far-UV wavelength range of 190–260 nm.

### 4.8. Post-Translational Modification Analysis by Mass Spectrometry

Each ADAMTS13 sample (20 µL; approximately 4 µg) was loaded onto a NuPAGE 4–12% gel (Thermo Fisher Scientific, Waltham, MA, USA) for electrophoresis. Single protein bands at approximately 190 kDa were observed for each sample using Coomassie staining. Each band was excised, destained, reduced, and carboxymethylated with DTT and iodoacetic acid and digested with Trypsin in 50 mM ammonium bicarbonate pH 8.4 for 14 h at 37 °C. Following extraction in 0.1% trifluoroacetic acid in acetonitrile, individual samples were reduced in volume and 0.1% formic acid was added for on-line LC-mass spectrometric analysis [31]. Aliquots from each of the two ADAMTS13 digests were analyzed by MS and CAD MS/MS using Q-TOF geometry technology [32]. Both XevoG2S (Waters) and QStar (Sciex) instruments were used, including on-line separation with respectively an Acquity LC microbore column (1 mm × 50 mm C-18) and a Dionex/LC Packings LC nanocapillary column (15 cm × 75 µm C-18). Interpretations of data were made by manual/visual inspection using well established fragmentation mechanisms [31,33].

### 4.9. Measuring Specific Activity—FRETS-VWF73 Assay

A fluorogenic FRETS-VWF73 substrate (Peptides International, Louisville, KY, USA), which contains the ADAMTS13 cleavage site flanked by fluorescent donor and quencher pairs, was used to determine ADAMTS13 specific activity essentially as described previously [64]. Substrate and purified protein samples were incubated separately at 37 °C for 5 min before combining. Forty-five fluorescence readings were taken at 1 min intervals at 37 °C by a Victor X3 plate reader (PerkinElmer, Waltham, MA, USA). Samples of purified protein containing equal amounts of total protein were assayed. ADAMTS13 activity was calculated as the change in fluorescence reading at 430 nm per min.

### 4.10. Modeling the Metalloprotease Domain and Its Docking with the DTCS Domains

The crystal structure of the DTCS domains of ADAMTS13 is available from the Protein Data Bank (PDB 3GHM). The metalloprotease domain of ADAMTS13 was built using comparative modeling in Robetta (confidence 0.99, http://robetta.bakerlab.org/) and compared to the I-TASSER server models (https://zhanglab.ccmb.med.umich.edu/I-TASSER/). Then, the top five modeled metalloproteases from Robetta with the lowest energies were first manually docked with the DTCS domain by using PyMOL (The PyMOL Molecular Graphics System, Version 4.5 Schrödinger, LLC) and using previously reported experimental results as guides to create a starting structure. The Rosetta Docking Protocol was then implemented in ROSIE (Rosetta Online Server, https://rosie.graylab.jhu.edu/) [65] to allow perturbations of the metalloprotease onto the DTCS domain. After manual inspection of the ten best-scoring structures from each of the runs, the ten best docked structures were analyzed further (Figure S7).

### 4.11. Statistical Analysis

All data are presented as mean ± standard deviation unless otherwise noted. Group means were compared by a Student’s *t*-test. For specific activity data, analysis was performed on log transformed fold change values. *p* value of <0.05 was considered statistically significant.

## Figures and Tables

**Table 1:** Relative synonymous codon usage (RSCU) values of prolineencoding codons in human genome, *ADAMTS13* cDNA, and liver tissue.

Variant	Codon	Relative Synonymous Codon Usage (RSCU) ^a^		
		Human Genome	*ADAMTS13* cDNA	Human Liver
WT	CCG	0.39	0.44	0.43
c.354G>A	CCA	1.19	0.92	1.03
c.354G>C	CCT	1.21	1.19	1.15
c.354G>T	CCC	1.21	1.46	1.39

^a^ RSCU values were calculated as described in methods.

**Table 2:** Codon pair usage (codon context) of prolineencoding codons in conjunction with preceding (GAC) and subsequent (TCC) codons at P-118 in *ADAMTS13* cDNA.

Variant	Codon Pairs	Human Genome		Human Liver	
		Codon Pair Frequency ^a^	Codon Pair Percentile	Codon Pair Frequency	Codon Pair Percentile
WT	GAC CCG CCG TCC	179.158 123.832	48.315 35.181	81.21 53.73	28.64 20.73
c.354G>A	GAC CCA CCA TCC	423.023 311.350	84.082 72.192	367.07 329.59	77.83 74.29
c.354G>C	GAC CCC CCC TCC	490.152 394.130	88.330 81.665	664.01 315.10	93.12 72.73
c.354G>T	GAC CCT CCT TCC	635.956 453.802	94.189 86.230	318.38 217.91	73.14 59.16

^a^ Codon pair frequency per million codon pairs.

**Table 3:** Summary of the O- and C-glycome findings for wild-type (WT) and p.P118P variant ADAMTS13.

Un-Mapped Signals Observed in the MS and MS/MS WT Data ^a^	Glycopeptide Assignments from the MS and MS/MS Spectra ^b^	Corresponding Signals Observed in p.P118P ^c^
601.2^2+^	^399^SJGGGVVTR^407^ + deoxyHex.Hex (TSR1)	601.2^2+^
765.8^2+^	^693^GPJSVSJGAGLR^704^ + deoxyHex.Hex (TSR2)	765.8^2+^
1380.0^4+^	^717^ELVETVQJQGSQQPPAWPEAJVLEPJPPYWAVGDFGS ASJGGGLR^763^ + deoxyHex.Hex (TSR3)	1380.0^4+^
857.0^3+^	^889^TGAQAAHVWTPVAGSJSVSJGR^910^+ deoxyHex.Hex (TSR5)	857.0^3+^
695.8^2+^	^959^LAAJSVSJGR^968^ + deoxyHex.Hex (TSR6)	695.8^2+^
1017.4^2+^	^1018^VMSLGPJSASJGLGTAR^1034^ + deoxyHex.Hex (TSR7)	1017.4^2+^
859.0^3+^	^1076^WHVGTWMEJSVSJGDGIQR^1094^ + deoxyHex.Hex (TSR8)	859.0^3+^
519.2^2+^	^387^WSSWGPR^393^ + Hex	519.2^2+^
1065.4^2+^	^1166^GLLFSPAPQPR^1176^ + NeuAc_2_HexHexNAc (major)	1065.4^2+^
832.2^3+^	^1166^GLLFSPAPQPR^1176^ + NeuAc_2_Hex_2_HexNAc_2_ (minor)	832.2^3+^

^a^ Signals in the MS and MS/MS data sets which did not map onto the expected ADAMTS13 tryptic peptide masses and were therefore prime candidates for Post-Translational Modification analysis and detailed interpretation. ^b^ Glycopeptide assignments (J = Carboxymethyl Cysteine) were made from a combination of the observed masses, known consensus motifs, inspection of b and y″ fragment ions in the MS/MS spectra to define sequence and (i) losses of 308.1 Da (Hex.DeoxyHex) from quasimolecular ions in the cases of TSRs, or (ii) losses of 138 Da and other characteristic ions shown in Figure S6 allowing the identification of Hexosyl W-387 and not W-390, or (iii) competitive losses of NeuAc, Hex, and HexNAc (NeuAc_2_HexHexNAc, DiSialyl Core-1, in total) in the case of S-1170. ^c^ The same observations were made for the p.P118P variant ADAMTS13, showing that the O- and C-Glycomes observed for both the samples are very similar.

## Supplementary Materials

Supplementary materials can be found at https://www.mdpi.com/1422-0067/20/22/5734/s1.

## Abbreviations

ADAMTS13	A disintegrin and metalloproteinase with a thrombospondin type 1 motif, member 13
DTCS	Disintegrin (D), thrombospondin type-1 repeat-1 (T), cysteine-rich (C), and spacer (S) domains
VWF	von Willebrand factor
RSCU	Relative Synonymous Codon Usage
PDB	Protein Data Bank
RIBO-SEQ	Ribosome Profiling
RNA-SEQ	RNA-Sequencing
HIVE-CUTS	High-performance Integrated Virtual Environment-Codon Usage Tables
CoCoPUTs	Codon and Codon-Pair Usage Tables
