# Supplementary material for: A Single Synonymous Variant (c.354G>A [p.P118P]) in ADAMTS13 Confers Enhanced Specific Activity
Source: Int J Mol Sci. 2019 Nov 15;20(22):5734. doi: 10.3390/ijms20225734 (PMC6888508; doi:10.3390/ijms20225734)
Supplement: Supplementary file 1 [file ijms-20-05734-s001.pdf]

Supplementary Figure Legends

Figure S1. *In Vitro* translation analysis of wild-type (WT) and c.354G>A variant *ADAMTS13* transcripts. Representative autoradiogram of WT and p.P118P (c.354G>A) variant *in vitro* translation products. ADAMTS13 protein is indicated by an arrow. Significantly higher translation of c.354G>A variant relative to WT transcript was observed. *In vitro* translation products of ADAMTS13 variants not included in current study are indicated.

Figure S2. Cumulative sum of (a) log (Ribosome Protected Fragment) *ADAMTS13* values. Cumulative sum of the normalized log (Ribosome Protected Fragments) of WT and c.354G>A (p.P118P) transcripts is shown. Data series for each construct represents two biological replicates of three technical replicates each.

Figure S3. Average normalized reads by codon number for *ADAMTS13* (WT and c.354G>A (p.P118P)), *ACTB* and *GAPDH*. Panels A and B respectively show ribosome profiles for *ADAMTS13* variants along the entire gene and focused around codon 118. Largely similar profiles were observed in both the plots. Panels C and D show ribosome profiles for control genes *ACTB* and *GAPDH* respectively. Data series for each construct represents two biological replicates of three technical replicates each.

Figure S4. Circular dichroism (CD) analysis of wild-type (WT) and p.P118P variants. CD spectra of WT and p.P118P variant proteins from three independent runs were displayed.

Supplementary Figure Legends (continued)

Figure S5. Glycosylation of W-387 in ADAMTS13. The MS/MS spectrum of m/z 519.2^{2+} showing the b and y′′ ions used to assign the sequence within wild-type ADAMTS13 as WSSWGPR. The quasimolecular ion is 162 Da, a Hexose unit, higher in mass than the theoretical peptide mass, and the position of substitution of the Hexose is on the first Tryptophan, W387, as proven by the interpretation of the fragment ions observed, shown in schematic with suggested mechanisms. There is no evidence of W-390 being Hexosylated. The principal fragment ions observed correspond to the novel formation of a 2-Ethynyl-Indole (Acetylenic substituent) on the side chain of W-387 resulting from loss of 138 Da due to water loss and partial cleavage of the Hexose ring, in preference to the normal β-elimination (162 Da) seen in O-linked glycosylation chemistry.

Figure S6. Glycosylation of S-1170 in ADAMTS13. The partial Mass Spectrum (m/z 550-1200) at the corresponding LC-MS elution time where the MS/MS of a signal at 1065.5^{2+} gave b and y′′ signals attributable to the sequence GLLFSPAPQPR in ADAMTS13. The spectrum shows an ion at 1065.5^{2+} and a corresponding quasimolecular ion for the peptide component of 1182.6 (591.8^{2+}) which calculates for a $NeuAc_2HexHexNAc$ unit attached to S1170 of the peptide sequence assigned. From a knowledge of mammalian biosynthetic pathways this likely corresponds to a DiSialyl Core-1 structure, and the sequential losses of individual sugar units observed (and annotated in the Figure) suggests the basic structure illustrated.

Figure S7. Rosetta Interface Score *vs* RMSD for the modelled metalloprotease docked on to the DTCS (Crystal structure of Disintegrin (D), TSR Repeat-1 (T), Cysteine-rich (C) and Spacer (S) domains of ADAMTS13) run. Each point represents a structure created by the ROSIE server with the top scoring structures shown in bold. The vertical axis gives the interface score (total score of the protein complex minus the total score of each partner in isolation, as described in the ROSIE documentation), while the horizontal axis gives the root-mean-square deviation (RMSD), a measure of distance from the starting position

Figure S1

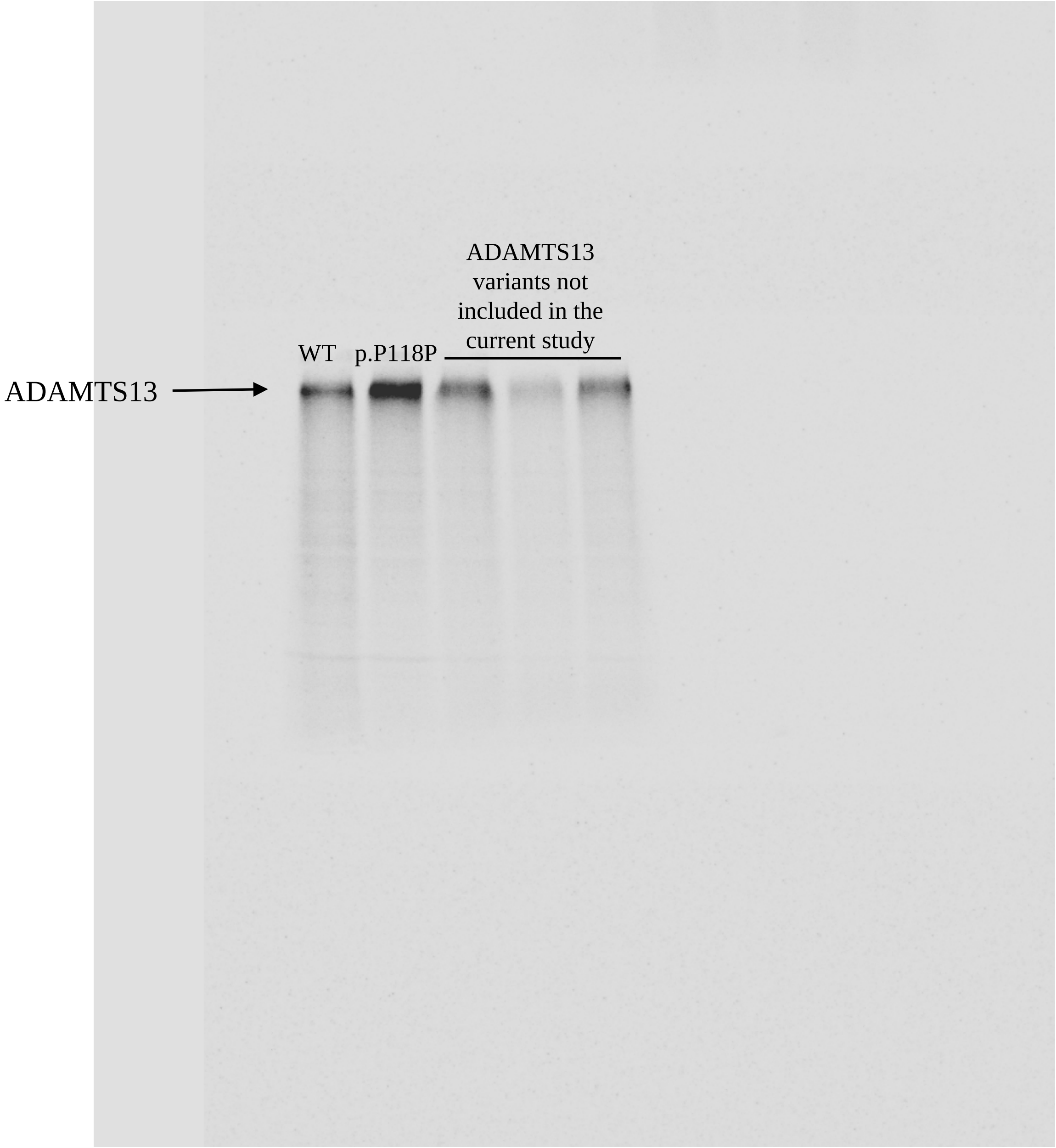
ADAMTS13 variants not included in the current study
WT
p.P118P
ADAMTS13

Figure S2

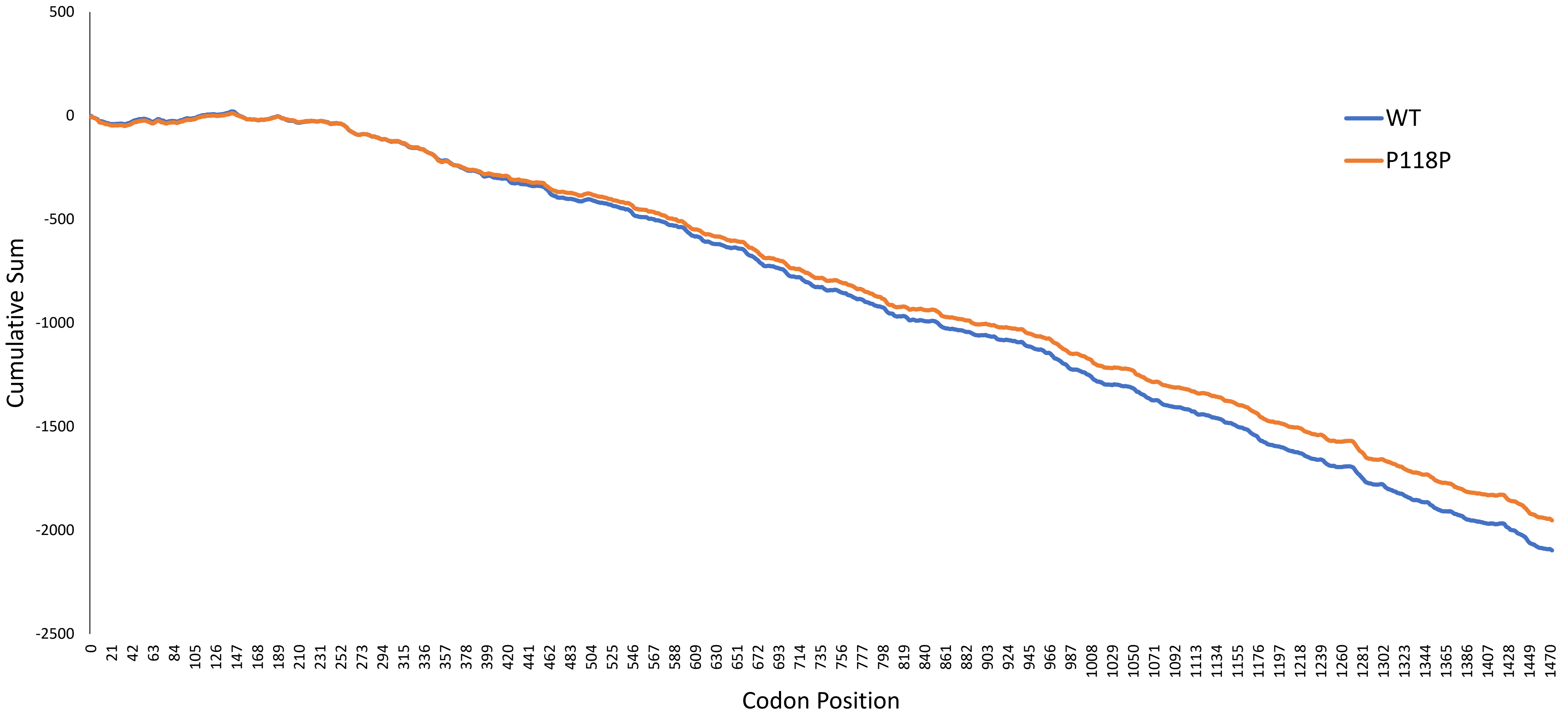
500
0
-500
-1000
-1500
-2000
-2500
Cumulative Sum
0 21 42 63 84 105 126 147 168 189 210 231 252 273 294 315 336 357 378 399 420 441 462 483 504 525 546 567 588 609 630 651 672 693 714 735 756 777 798 819 840 861 882 903 924 945 966 987 1008 1029 1050 1071 1092 1113 1134 1155 1176 1197 1218 1239 1260 1281 1302 1323 1344 1365 1386 1407 1428 1449 1470
Codon Position
WT
P118P

Figure S3

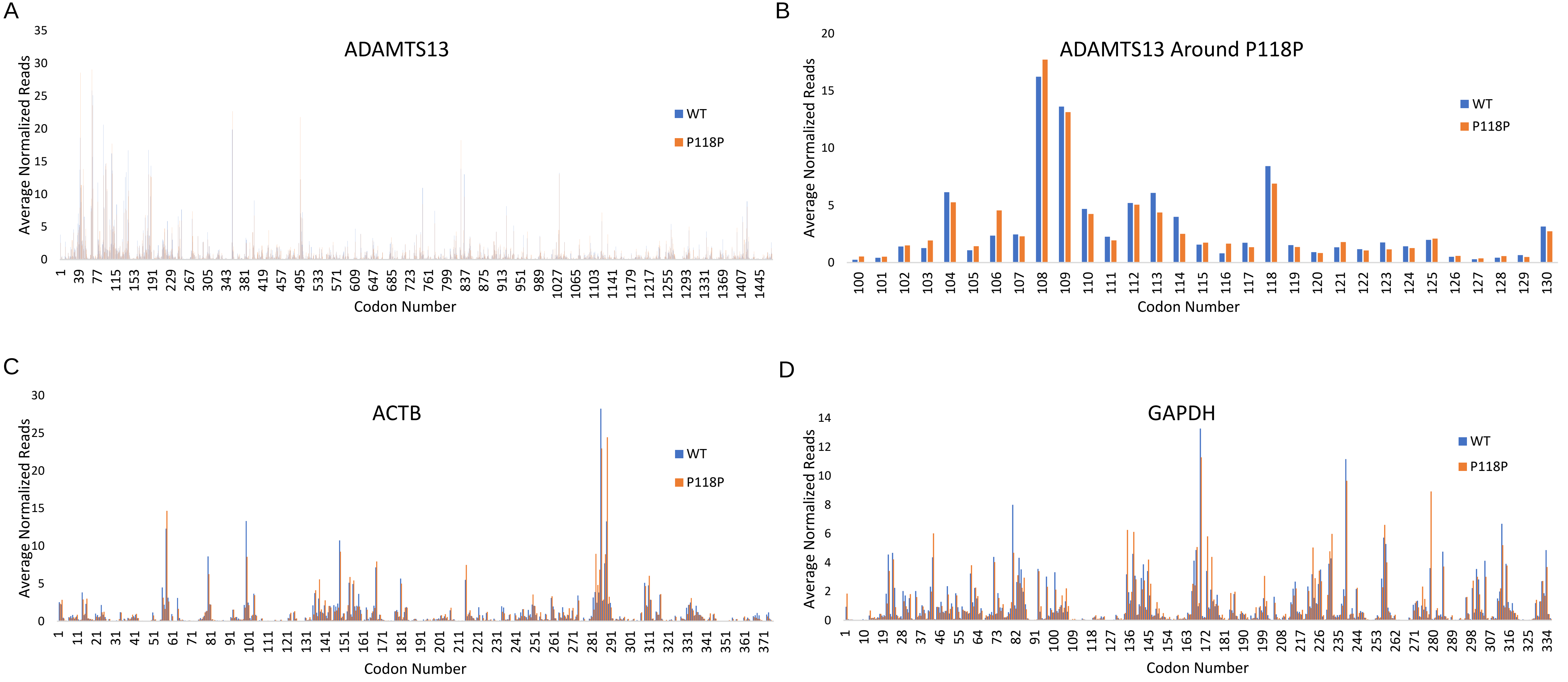
A
ADAMTS13
Average Normalized Reads
Codon Number
WT
P118P
B
ADAMTS13 Around P118P
Average Normalized Reads
Codon Number
WT
P118P
C
ACTB
Average Normalized Reads
Codon Number
WT
P118P
D
GAPDH
Average Normalized Reads
Codon Number
WT
P118P

Figure S4

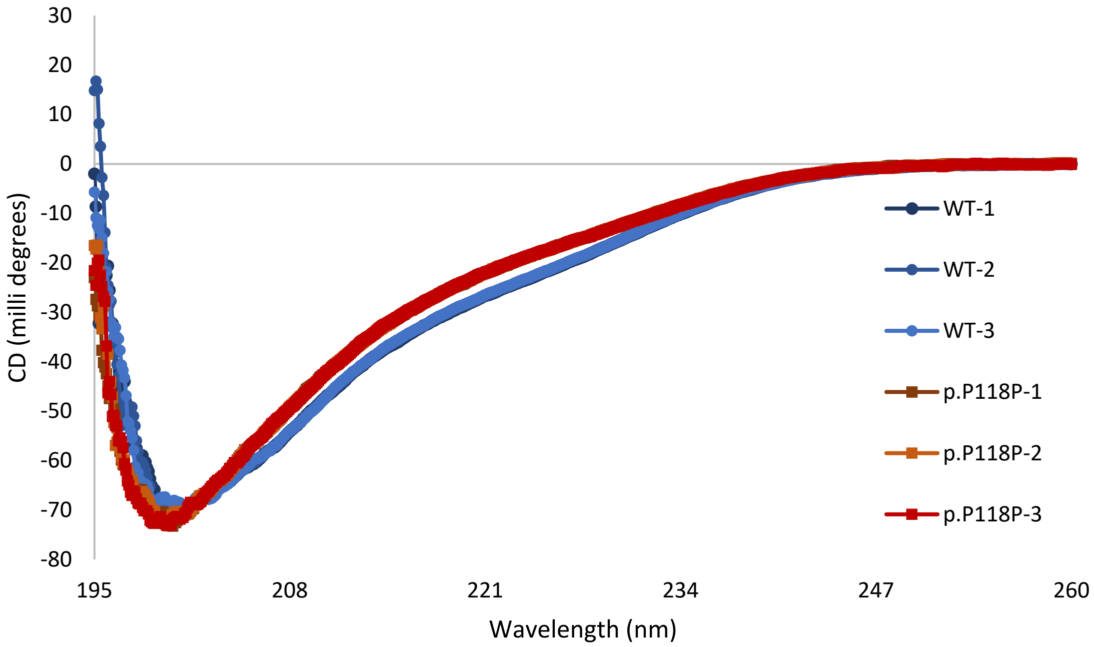
30
20
10
0
-10
-20
-30
-40
-50
-60
-70
-80
CD (milli degrees)
195
208
221
234
247
260
Wavelength (nm)
WT-1
WT-2
WT-3
p.P118P-1
p.P118P-2
p.P118P-3

Figure S5

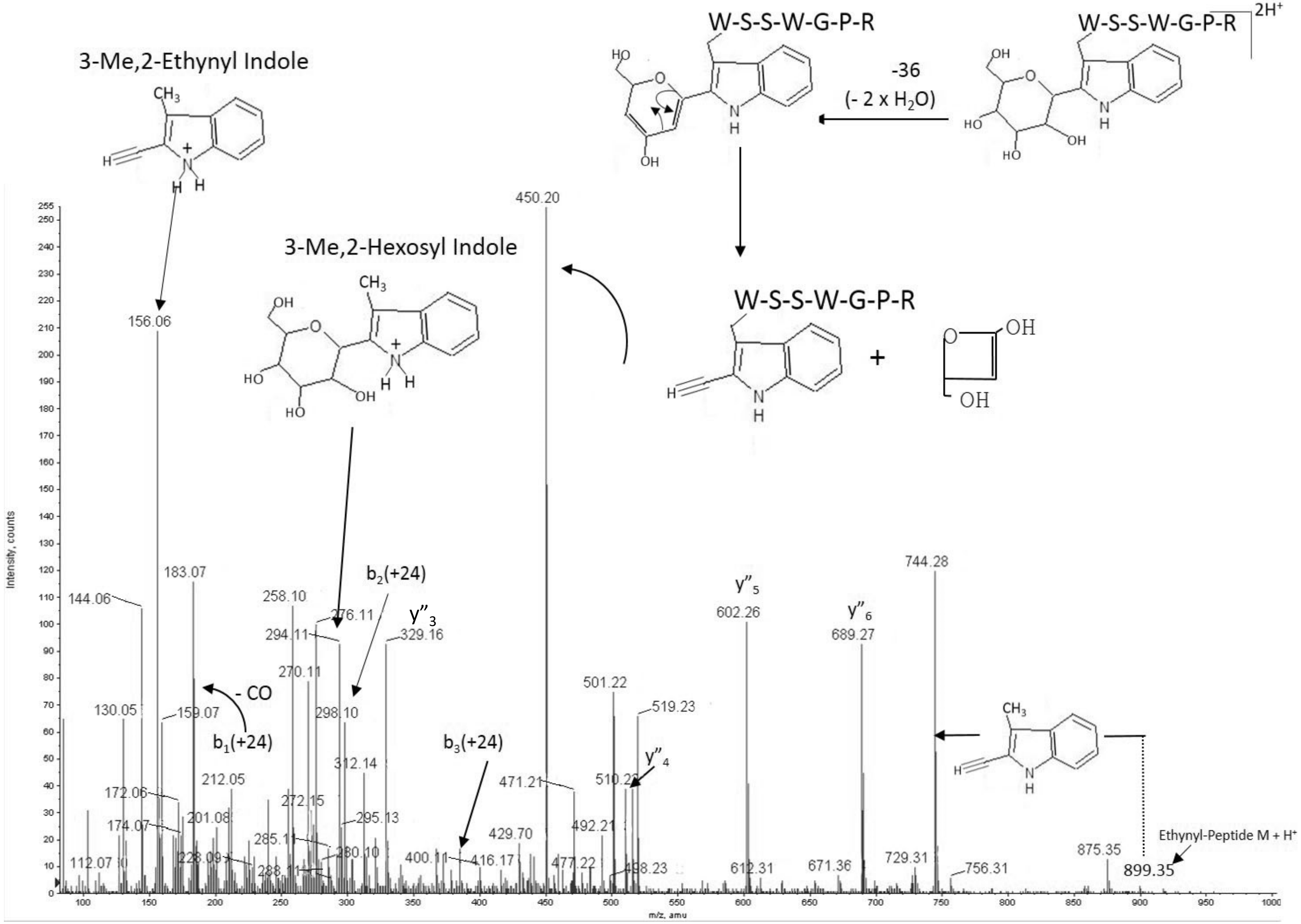

3-Me,2-Ethynyl Indole
3-Me,2-Hexosyl Indole
W-S-S-W-G-P-R
2H+
-36
(- 2 x H2O)
+
450.20
156.06
183.07
144.06
258.10
276.11
294.11
329.16
270.11
298.10
130.05
159.07
- CO
b1(+24)
b2(+24)
b3(+24)
y"3
y"4
y"5
y"6
312.14
212.05
172.06
272.15
295.13
201.08
174.07
285.11
280.10
228.09
288.11
112.07
400.11
416.17
429.70
471.21
477.22
492.21
501.22
510.22
519.23
498.23
602.26
612.31
671.36
689.27
729.31
744.28
756.31
875.35
899.35
Ethynyl-Peptide M + H+
Intensity, counts
m/z, amu

Figure S6

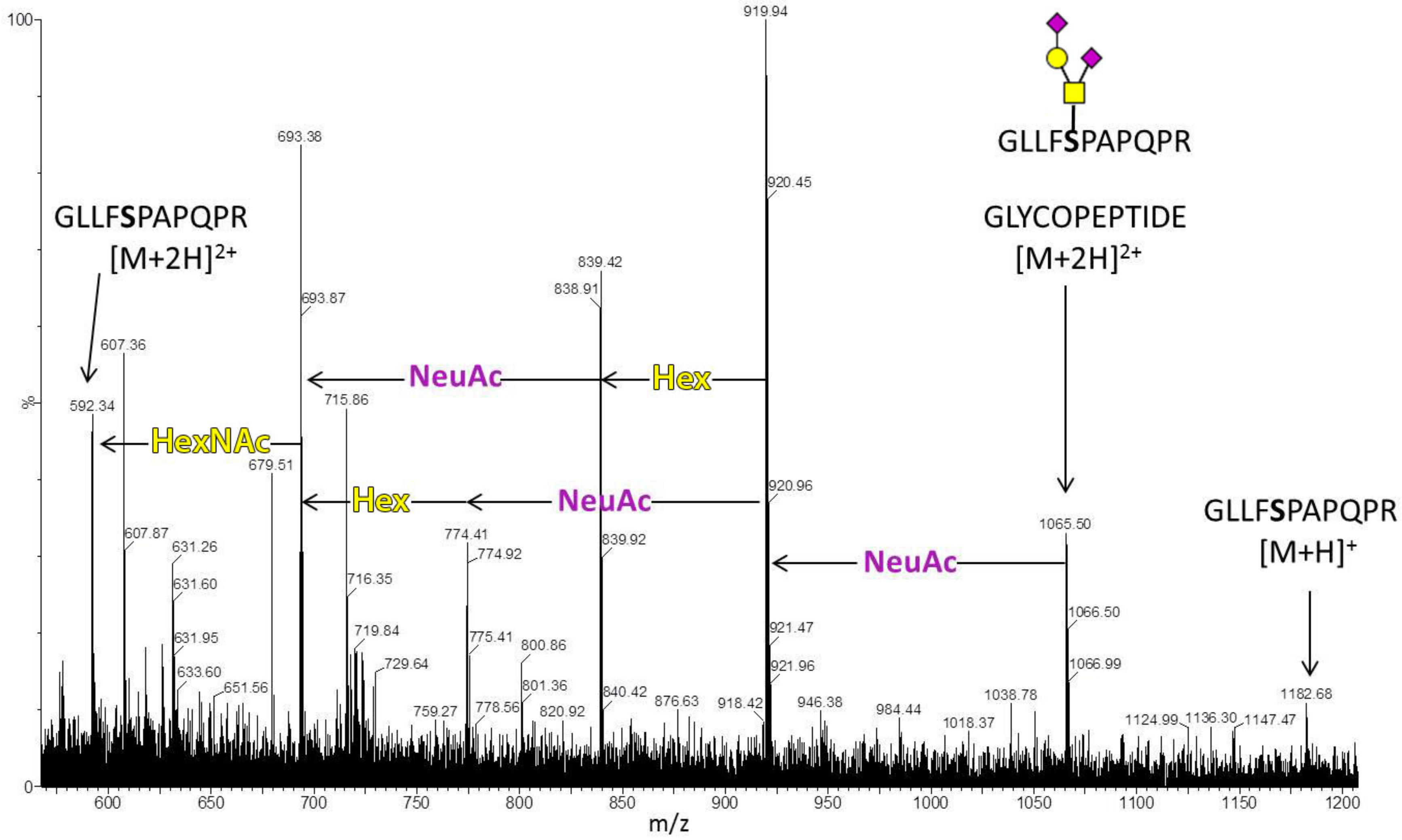
GLLFSPAPQPR
GLYCOPEPTIDE
[M+2H]2+
GLLFSPAPQPR
[M+2H]2+
GLLFSPAPQPR
[M+H]+
NeuAc
Hex
HexNAc
Hex
NeuAc
NeuAc
919.94
920.45
920.96
921.47
921.96
918.42
693.38
693.87
839.42
838.91
839.92
840.42
607.36
607.87
592.34
715.86
716.35
719.84
729.64
679.51
774.41
774.92
775.41
778.56
759.27
800.86
801.36
820.92
631.26
631.60
631.95
633.60
651.56
876.63
946.38
984.44
1018.37
1038.78
1065.50
1066.50
1066.99
1124.99
1136.30
1147.47
1182.68
100
%
0
m/z

Figure S7

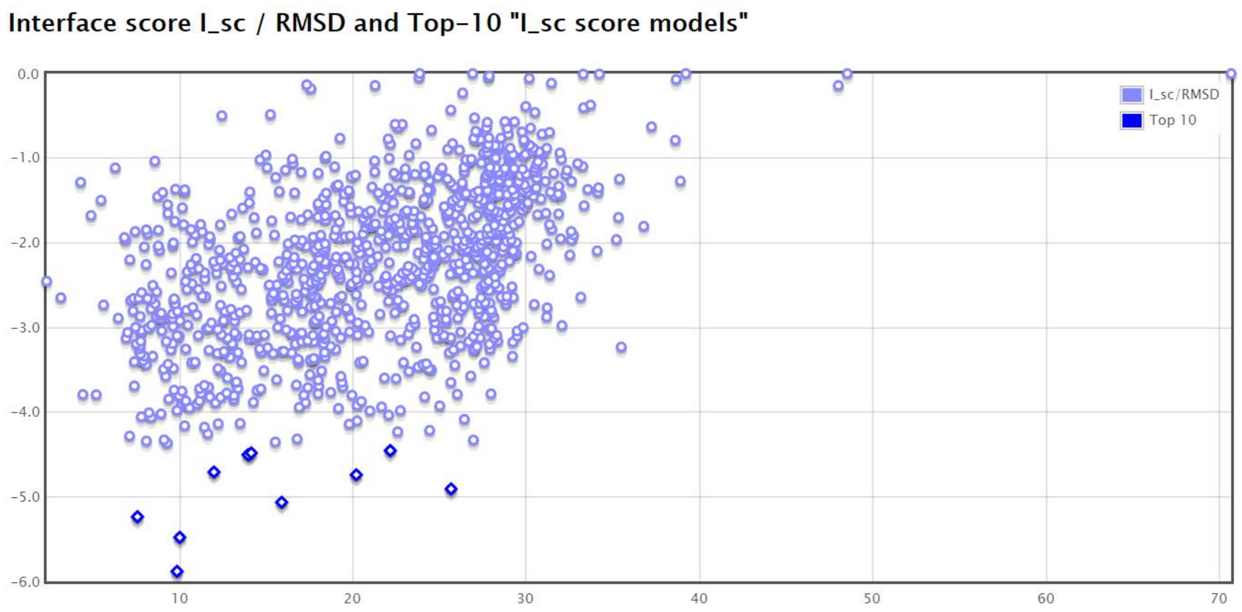

Interface score I_sc / RMSD and Top-10 "I_sc score models"
I_sc/RMSD
Top 10
0.0
-1.0
-2.0
-3.0
-4.0
-5.0
-6.0
10
20
30
40
50
60
70
